# Somatotopic Mapping of the Fingers in the Somatosensory Cortex Using Functional Magnetic Resonance Imaging: A Review of Literature

**DOI:** 10.3389/fnana.2022.866848

**Published:** 2022-06-29

**Authors:** Daniel Janko, Kristina Thoenes, Dahye Park, W. R. Willoughby, Meredith Horton, Mark Bolding

**Affiliations:** ^1^Department of Psychology, University of Alabama at Birmingham, Birmingham, AL, United States; ^2^Department of Neurology, University of Alabama at Birmingham, Birmingham, AL, United States; ^3^Edward Via College of Osteopathic Medicine Auburn, Auburn, AL, United States; ^4^School of Medicine, University of Alabama at Birmingham, Birmingham, AL, United States; ^5^Department of Radiology, University of Alabama at Birmingham, Birmingham, AL, United States

**Keywords:** finger somatotopy, neuroimaging, fMRI, digit overlap, digit distance, somatosensory cortex, cortical magnification

## Abstract

Multiple studies have demonstrated finger somatotopy in humans and other primates using a variety of brain mapping techniques including functional magnetic resonance imaging (fMRI). Here, we review the literature to better understand the reliability of fMRI for mapping the somatosensory cortex. We have chosen to focus on the hand and fingers as these areas have the largest representation and have been the subject of the largest number of somatotopic mapping experiments. Regardless of the methods used, individual finger somatosensory maps were found to be organized across Brodmann areas (BAs) 3b, 1, and 2 in lateral-to-medial and inferior-to-superior fashion moving from the thumb to the pinky. However, some consistent discrepancies are found that depend principally on the method used to stimulate the hand and fingers. Therefore, we suggest that a comparative analysis of different types of stimulation be performed to address the differences described in this review.

## Introduction

Using a variety of brain mapping methods, multiple studies have been conducted in humans and non-human primates to examine the somatotopy of different parts of the body. The majority of the studies have examined hand and finger somatotopy. Brain mapping methods included intraoperative electrodes (Roux et al., 2018), electrocorticography (eCoG) (Sutherling et al., 1992), intraoperative evoked potentials (Woolsey et al., 1979), positron emission tomography (PET) (Fox et al., 1987; Hagen and Pardo, 2002), magnetoencephalography (MEG) (Baumgartner et al., 1991; Cheyne et al., 2000; Inoue et al., 2013), electroencephalography (EEG) (Baumgartner et al., 1993; Cheyne et al., 2000), and functional magnetic resonance imaging (fMRI) (Maldjian et al., 1999; Kurth et al., 2000; Hlustík et al., 2001; Overduin and Servos, 2004; Nelson and Chen, 2008; Sanchez-Panchuelo et al., 2010; Stringer et al., 2011; Besle et al., 2013; Martuzzi et al., 2014; Sánchez-Panchuelo et al., 2014; Kolasinski et al., 2016; Sanchez Panchuelo et al., 2018; Schellekens et al., 2018, 2021; Puckett et al., 2020; Willoughby et al., 2020; Arbuckle et al., 2021; Wang et al., 2021). Regardless of the method used, individual somatosensory maps were consistently organized in BA 3b in a lateral-to-medial and inferior-to-superior fashion moving from the thumb to the little finger.

Given the ubiquity of fMRI in human brain research, there is a significant motivation to evaluate the consistency of fMRI brain mapping results across studies. The objective of this review is to examine the results of somatotopic mapping of the hand and fingers in the somatosensory cortex using fMRI. We intend to focus on the similarities and differences reported in fMRI studies to give an overview of the localization of the finger somatotopy and the reported overlap between adjacent fingers. This review is not a quantitative meta analysis of the literature.

### Human Somatosensory Cortex

**FIGURE 1:**
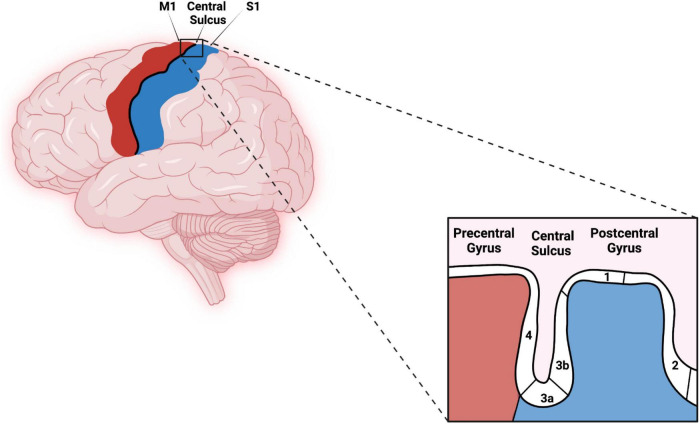
Original work showing lateral view of the primary motor cortex, central sulcus (CS), primary somatosensory cortex, and corresponding Brodmann areas (BAs) (4, 3a, 3b, 1, and 2). Red–Primary motor cortex, Blues–Primary somatosensory cortex. The figure was created using Biorender.com.

The human somatosensory cortex consists of BAs 1, 2, 3a, and 3b. These areas extend across the central sulcus (CS) and postcentral gyrus (PG) with BA 3a located in the fundus of the CS. BA 3b is located in the rostral bank, BA 1 in the crown, and BA 2 in the caudal bank of PG as shown in Figure 1 (Geyer et al., 1999, 2000). Some investigators suggest that BA 3b should be considered the primary somatosensory cortex (S1) for processing tactile information (Kaas et al., 1979; Purves, 2001).

The modern understanding of the somatosensory cortex began with the study performed by Penfield and Boldrey (1937). Intraoperative electrode stimulation was used in awake patients to create a map of the somatosensory cortex. Each stimulation point was related to a corresponding body location based on the report of the patient. They discovered that sensation in the fingers constituted over one-sixth of the total surface area of the primary somatosensory cortex. They also noted that this area extended along the anterior edge of the post central gyrus, adjacent to the CS. Based on these findings, Penfield and Boldrey formulated a map of the primary somatosensory cortex known as the *sensory homunculus*. The sensory homunculus topographically represents the sensory distribution of each body part based on the area of stimulation and the corresponding sensory locations reported by patients. Specifically, different body parts occupy different regions of the brain along the PG (Nguyen and Duong, 2021). Crucially, they found that the size of a body region in the map does not depend on the actual physical size of the body part, but rather on its sensitivity, a phenomenon referred to as cortical magnification. Hands, lips, and tongue were associated with the greatest number of stimulation points. That is, the body areas with the most sensitivity extend over the largest cortical area (Penfield and Boldrey, 1937). Corniani and Saal (2020), confirmed that there are differences between the density of innervation by tactile afferents that correspond to the differences in sensitivity of different parts of the body.

### Tactile Signals

The human hand is innervated by multiple types of cutaneous and subcutaneous mechanoreceptors. Meissner’s corpuscles respond to dynamic skin deformation and low-frequency vibration. Pacinian corpuscles are sensitive to high-frequency stimuli and Ruffini corpuscles are responsive to low-frequency vibration or pressure. Lastly, Merkel’s discs are sensitive to light touch and transmit spatial structure of objects. The combined activation of all four receptors creates the sensation of touch (Johnson et al., 2000; Willis and Westlund, 2001; Raju and Tadi, 2020; Harrow-Mortelliti et al., 2021). When an external stimulus meets the required threshold intensity and is detected by these receptors, the action potentials are propagated through the dorsal column pathway in the spinal cord to the somatosensory cortex where the touch is processed as sensation. The ascending pathway of the dorsal column tract carries its electrical signal through three distinct neurons before reaching the parietal lobe and primary somatosensory cortex (Al-Chalabi et al., 2022). Though the forms of stimulation differ across the studies reviewed, each study stimulated mechanoreceptors that are the first-order neurons in the ascending sensory tract.

### Different Experimental Designs Used in Functional Magnetic Resonance Imaging Somatotopy Studies

**FIGURE 2:**
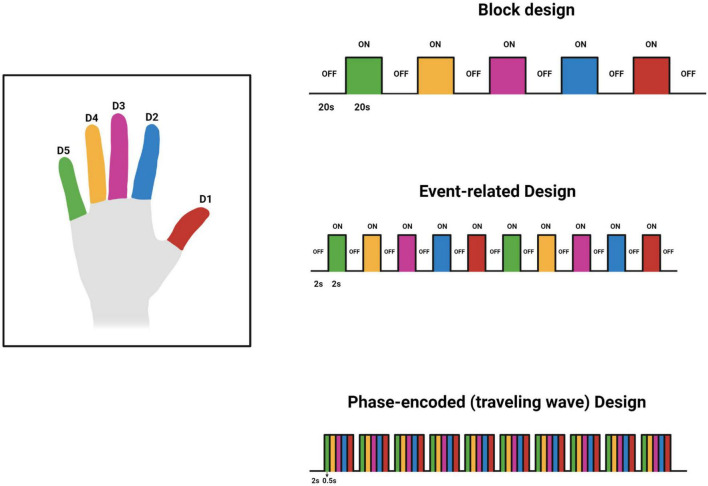
Original work showing 3 designs used for investigating the finger somatotopy. The timings in the figure are tentative and may vary based on the investigators’ preferences and other factors. This figure was created using Biorender.com.

Various experimental designs have been used to investigate the BOLD response associated with tactile stimuli. These designs are predominantly block, phase-encoding, and event-related designs. Block design fMRI studies have sequential uniform time blocks of stimulus and rest. These blocks alternate and their duration results in summation of the BOLD signal to increase detectability (Kannurpatti et al., 2011). For example, as shown in Figure 2, 20 s of rest is followed by 20 s of stimulus. The blocks are generally repeated several times to improve the estimate of the response to the task or stimulus. Event-related fMRI designs employ brief stimuli compared to block designs (Kannurpatti et al., 2011). This allows for more efficient randomization and for estimation of the hemodynamic response to single events. A phase-encoding design uses a periodic stimulation varying over space or some other continuous parameter such as audio frequency to estimate the amplitude and phase of a hemodynamic response across a region of the brain such as primary visual cortex or primary auditory cortex (Engel, 2012). This design is often used to generate cortical maps of pRF s such as the retinotopic maps in visual cortex. The designs used by each reviewed study can be found in Tables 1, 2. For an excellent in-depth introductions to fMRI design and analysis see Huettel et al. (2008) and Poldrack et al. (2011).

**TABLE 1:** Overview of fMRI studies.

Author/s	(Kolasinski et al., 2016)	(Maldjian et al., 1999)	(Martuzzi et al., 2014)	(Nelson and Chen, 2008)	(Overduin and Servos, 2004)	(Puckett et al., 2020)	(Sanchez-Panchuelo et al., 2010)	(Sánchez-Panchuelo et al., 2014)
Phalange/digits stimulated	D2–D5	D1–D5 finger pad	D1–D5 two distal phalanges of each finger	D1–D5	Thumb, index, and ring finger	Distal phalanx (D2–D4)	Digit tips	Proximal-distal phalanges (D2–D4)
Distance D1–D5	Average distances: D2-D3 = 10.24 mm; D3–D4 = 7.27 mm; D4–D5 = 6.30 mm	18 mm between D1 and D5. Area not specified	15.5 ± 2.4 mm in BA3b; 15.1 ± 4.3 mm in BA 1; 8.6 ± 4.2 mm in BA 2	Distances from D1 to D5 in Brodmann Areas: 3b = 17.9 mm, 1 s = 14.9 mm, 1i = 4.4 mm, 2 = 6.8 mm	Not reported	Not reported	Not reported	Not reported
Design	Phase-encoding design	Block design	Block design	Block design	Event-related design	Phase-encoded design	Traveling wave and event-related designs	Phase-encoded design
Number of subjects Hand	13 Right hand (dominant)	5 Right hand	10 Right hand	12 Right hand	6 Right hand	6 Not specified	5 Left hand	4 Left hand
Stimulation type Area stimulated	Motor task	Vibrotactile stimulation (15–30 Hz) Not specified	Tactile stimulation (touch, 1 Hz) Not specified	Vibrotactile stimulation (23 Hz) ∼7 mm^2^	Air Tactile stimulation (10 Hz) Not specified	Vibrotactile (5, 20, 100 Hz) Entire pad of each digit	Vibrotactile stimulation (30 Hz) 1 mm^2^	Vibrotactile (30 Hz) 1 mm^2^
Stimulation timing	8 s phases–8 cycles of 1 Hz finger movement–two movements per finger	The stimulus was delivered using a 20 s on, 20 s off paradigm, with the vibrational frequency alternating between 15 and 30 Hz every 5 s during the on period.	Each digit was independently stroked for 20 s, followed by 10 s of rest (no stroking	Consisted of 10 cycles of alternating periods of no vibration (22 s) and vibration (8 s)	6 cycles of 36 s stimulation, each separated by 60 ms	7.872 s for each finger per cycle; 5 cycles	Traveling wave: each digit stimulated 3 s with an off period of 1.8 s Event related: all five digits stimulated simultaneously for an on period of 3 s with random interstimulation intervals of 18,19, or 20 s.	16 0.4 s stimulation windows with 0.1 s gaps per cycle; 8 cycles
Cortical magnification	Not reported	Index finger and thumb displayed larger activation volumes than remaining fingers	Thumb had significantly larger representation in BA1 and BA2 and showed a trend toward larger magnification in BA3b.	Found that adjacent digit maps could not be separated in 3-dimensional space	Thumb represented the most and ring finger bands the least.	D2 had significantly larger cortical representation compared to other studied digits	Smaller phase delays for digits 1 and 2 and increasingly larger for remaining digits. No other cortical magnification discussed	Not reported
Field strength fMRI Data analysis Voxel size	7T MRI GLM Spatial smoothing using Gaussian kernel Voxel size: 8 mm^3^	4T MRI Data smoothing applied *via* FWHM Gaussian smoothing kernel. No smoothing Effective voxel resolution: 1.875 mm × 1.875 mm × 3 mm or 10.55 mm^3^	7T MRI BOLD response represented by relevant GLM regressors Smoothened with an isotropic Gaussian kernel (FWHM = 2 mm). Voxel Resolution: 1.3 mm × 1.3 mm × 1.3 mm	3T MRI No specific analysis models mentioned No smoothing Voxel resolution: 10 mm^3^	4T Talairach standardization No smoothing Voxel size: 1.5 mm × 1.5 mm × 1.5 mm	7T MRI GLM + population receptive fields modeling Smoothened Voxel resolution: 0.8 mm × 0.8 mm × 0.8 mm	7T MRI No spatial filtering applied. Image-based shimming to reduce geometrical distortion. Voxel Resolution: 1 mm^3^	7T MRI Structural images were smoothened Voxel resolution: 1.25 mm × 1.25 mm × 1.25 mm
Main findings	“Observed locations of digit somatotopy were consistent with BA 3b. Highly reproducible maps of individual digits in S1. Significant variability among subjects in the shape, extent, and positioning of digit representation. Raising the question of population variability.”	“Lateral-to-medial, inferior-to-superior, and anterior-to-posterior organization from the thumb to the fifth finger. Considerable overlap is seen between digits.”	“Little finger localized to a more superior and medial position and thumb to a more inferior and lateral region. Found overlap between fingers and less finger-specific maps in BAs 1 and 2.”	“Anterior-posterior dissociation of adjacent finger was not possible Less degree of territory dedicated to each finger in Area 1 compared to 3b Not designed to investigate the overlap between adjacent fingers.”	“Area 3b had a greater fraction of observed SI phase bands, indicating that it receives input from two receptor types (Meissener and Merkel’s).”	“Finger representation was found along the postcentral gyrus. pRFs of the little finger were bigger than pRFs of other studied fingers. Size of pRF increases moving posterior in the postcentral sulcus.”	“Orderly map of the digits on the posterior bank of the central sulcus. Activation of the digits falls nearly entirely along the posterior central sulcus and anterior superior postcentral gyrus. Lateral to medial and inferior to superior organization of the digits from thumb to the little finger.”	“Finger representation was found in all four araas (3a, 3b, 1, 2). Cortical thickness increases moving posterior in S1.”

**TABLE 2:** Overview of fMRI studies.

Author/s	(Sanchez Panchuelo et al., 2018)	(Schellekens et al., 2018)	(Schellekens et al., 2021)	(Schweisfurth et al., 2014	(Schweisfurth et al., 2018)	(Schweizer et al., 2008)	(Stringer et al., 2011)	(Wang et al., 2021)
Phalange/digits stimulated	D1–D5	Whole finger	Fingertips	All phalanges (D1–D5)	Distal phalanges (D1–D5)	First phalanx (D1–D5)	Distal phalanx (D1–D5)	2 phalanges at a time (D1–D5)
Distance D1–D5	Not reported	Not reported	Not reported	Increasing Euclidean distance between D1 and other digits moving medially.	Distance D1–D5–left hand = 18 ± 2 mm, right hand = 14 ±4 mm	Not reported	D1–D5 in 3b = 12 mm (surface), 7.46 mm (Euclidean) D1–D5 in 1 = 7.25 mm (surface), 4.68 mm (Euclidian)	Significantly increasing distance moving from D1 to D5
Design	Phase-encoded design + Block design	Event-related design	Event-related design	Block design	Block design	Block design	Block design	Phase-encoded design
Number of subjects Hand	6/4 Left hand	8 Right hand	8 Right hand	18 Right hand (dominant hand)	12 Both hands	6 Right hand	6 Not specified	10 Left hand (non-dominant)
Stimulation type Area stimulated	Vibrotactile stimulation (30 Hz) 1 mm^2^	Flexion and Extension Whole finger	Vibrotactile stimulation (30 Hz, 110 Hz, and 190 Hz) 1 mm^2^	Tactile (32 Hz) 18.75 mm^2^	Mechanical tactile (32 Hz) 18.75 mm^2^	Tactile stimulation (16 Hz) 18.75 mm^2^	Air puffs (2 Hz) Not specified	Mechanical tactile (150 mN) Not specified
Stimulation timing	5 s on/19 s off, 8 s on/22 s off, or 14 s on/26 s off; 60 s 3 × each digit	Cued movement (extension/flexion) followed by a 4.8 s gap before the next finger	400 ms 100 ms off for 4 s = 1 cycle per cycle; 8 cycles; 3 runs	Between digits: 12 s on/12 s off each digit stimulated 5 × Within digits: 12 s on/12 s off distal phalanx stimulated 1 × and other 2 stimulated 2 ×	12 s on and 12 s off–each digit stimulated 8 times in total	1st run: 12 s on/12 s off 7 cycles; 2nd run: 18 s on/18 s off 20 cycles	6–12 of 24 s stimulation runs for each digit	700 ms on 300 ms off–8 cycles for each location–8 s for each location in total
Cortical magnification	Not reported	Not reported	pRF sizes are smallest for thumb representations and gradually increased for the remaining 4 fingertips.	Not reported	Thumb representation had larger volumes	Neuronal representation of the little finger is the smallest.	Greater magnification factor (M) in 3b than in 1.	Did not find significantly larger cortical magnification for any individual finger
Field strength fMRI Data analysis Voxel size	7T MRI Threshold *P* < 0.01 Maps generated *via* voxel-wise fitting with GLM Voxel Resolution: 1.5 mm^3^	7T MRI Gaussian population receptive fields model No smoothing Voxel resolution: 0.49 mm × 0.49 mm × 0.8 mm	7T MRI Gaussian population receptive fields model No smoothing Voxel resolution: 1.6 mm^3^	3T MRI GLM No smoothing Voxel sixe: 1.5 mm × 1.5 mm × 1.5 mm	3T MRI No spatial smoothing GLM Voxel size: 1.5 mm × 1.5 mm × 1.5 mm	3T MRI GLM No smoothing Voxel resolution: 1 mm^3^	7T MRI No spatial smoothing GLM Single- subject analysis Voxel size: 1 mm^3^	7T MRI Linear correlation No smoothing Voxel size = 0.7 mm × 0.7 mm × 0.7 mm
Main findings	“Mediolateral and superior to inferior axis from Digit 5 to 1. Greater specificity (less overlap) is seen for SI compared to SII digit representations.”	“An orderly representation of the fingers in the primary somatosensory cortex. Little finger showed the largest pRF compared to other digits.”	“pRF sizes increase with increasing frequency of vibrotactile stimulation in BA 1,2, and 3. pRFs increase moving from BA3b to BA2. Somatotopy in BA2 is less clear than in BA1 and BA3b.”	“Found an orderly representation of the fingers after stimulating the first phalanx in BA3b. The same was shown for both of the other phalanges (medial and proximal) as well. Only D5 within-finger maps showed a significant consistency across subjects.”	“No general difference between the digit representation of the left and the right hand can be determined.”	“Digit representations were found almost exclusively in BA 3b within S1 in medial to lateral (D5 to D1). Very limited overlap found in BA3b.”	“Found a clear separation of adjacent digits within areas 3b and 1. The results suggest an important role of BA3b on the representation of tactile acuity based on the magnification factor.”	“Orderly representation Puckett et al. (2020) of D1–D5 in the postcentral gyrus. individual pRF were larger in BA1 and BA2 than in BA3b suggesting more spatial overlap between digits in the more posterior areas.”

Besle et al. (2013) studied differences between event-related and phase-encoding designs and which of these two designs is more suitable for specific research questions. They found that both designs showed similar specificity maps and the main representation in S1 was clearly visible regardless of parameters. In order to obtain adequate maps, phase-encoding design only required 2 runs of 6 min while the event-related necessitated 6–8 4 min runs. This finding shows that phase-encoding design is more efficient, requiring less time in the scanner while still achieving the same desired results. However, when studying overlapping cortical response and overlap in general, event-related design provided additional information and helped with interpretation of specific features of phase-encoding maps (Besle et al., 2013).

## Literature Overview

Twenty one fMRI studies were reviewed to study the finger somatotopy in the primary somatosensory cortex. Overview of each study’s specification are shown in Tables 1, 2. Studies included in this review used either passive tactile/vibrotactile stimulation or active movements to stimulate the fingers with the exception of Kurth et al. that used electrical stimulation. As shown by Sanders et al. (2019) passive and active stimulations elicited similar responses in S1 and thus, they can be considered as a reliable stimulation for somatosensory mapping. Several different field strengths were used across the studies. Twelve studies used 7T, four studies used 3T, two studies used 4T, and the weakest field strength used was 1.5T in 3 studies. In general, higher field strengths result in better signal to noise and better spatial resolution in human fMRI studies. A caveat is that some areas, such as the orbitofrontal cortex, may be affected more by magnetic susceptibility artifacts at higher field strengths. For the cortical areas considered here, this should not be a concern.

## Results

The findings of Arbuckle et al. (2021) indicated somatotopic interactions between adjacent fingers in BA 3b. Regions located more anterior and posterior to area 3b showed less finger specificity and more overlap between individual fingers during single-finger stimulation. Additionally, medial-to-lateral and anterior-to-posterior organization of the fingers were observed (Arbuckle et al., 2021).

Besle et al. (2013) focused specifically on quantification of spatial overlap between adjacent fingers. Medial-lateral and anterior-posterior organization of the fingers were observed in S1 and this study showed a significant increase in overlap moving from BA 3b posteriorly to BAs 1 and 2. Importantly, their studies suggest that using event-related design is more effective for investigation of the overlap between adjacent fingers (Besle et al., 2013).

While Gelnar et al. (1998) failed to find a simple, medial-to-lateral arrangement of digits 1, 2, and 5, this study demonstrated the greatest distance between digits 1 and 5. Statistically significant differences between each stimulated digit were seen, suggesting that the response observed in cortex to the same stimulus may vary with the area of cortex being studied and the digit stimulated (Gelnar et al., 1998).

Hlustík et al. (2001) found an orderly somatotopy in both primary motor cortex and primary somatosensory cortex. Both areas showed significant overlap for individual digits during separate movements, but M1 showed more overlap. S1 activation was distributed into more clusters. They also found a trend for more S1 volume being activated during sequential movements compared to single digit movements (Hlustík et al., 2001).

Kurth et al. (2000) reported a larger overlap of adjacent digits as well as non-adjacent digits in BA1 and BA2 compared to BA3b. Furthermore, BA3b was activated with less contribution from BA1 and B2, and a rare activation in BA3a was also evident. This study also demonstrated that the average extension of fingers over S1 was 16 mm (Kurth et al., 2000).

Kolasinski et al. (2016) demonstrated a rather small intrasubject variability compared to a larger large intersubject variability in the distribution of individual finger representations. This calls into question the similarity of the somatosensory maps between individuals. They also reported overlap between individual fingers in the somatosensory cortex with more overlap and less finger sensitivity in BAs 1 and 2 (Kolasinski et al., 2016).

Maldjian et al. (1999) showed lateral-to-medial, inferior-to-superior, and anterior-to-posterior organization of the five fingers (D1–D5) in the somatosensory cortex. The spatial distance between D1 and D5 was found to be 18 mm in the primary somatosensory cortex. They also noted a considerable overlap between fingers, mostly between D5 and D2 and D5 and D3 (Maldjian et al., 1999).

Martuzzi et al. (2014) found an orderly somatotopy of the fingers in BAs 3b, 1, and 2. Overlap between adjacent fingers was shown for D4-D5 in BA3b. BA3b appeared to be more finger specific as the BOLD signal decreased when other digits were stimulated in this area. However, moving posterior from BA3b, a stronger cross-finger response was noted in BA 1 and 2. All areas of S1 showed inferior-to-superior organization with the thumb being most inferior and pinky most superior (Martuzzi et al., 2014).

Nelson and Chen (2008) found that stimulation of individual digits activated all areas of S1. Based on their findings, they subdivided BA1 into BA1–superior (BA1s) and BA1- inferior (BA1i). The total distance between thumb (D1) and pinky (D5) differed across the discrete areas defined in this study with BA3b having the largest space and BA1 having the smallest space. Moving posterior from BA3b, they reported a decrease in finger specificity and more overlap between fingers. BA2 did not include any regions specific to individual digits and the topographic organization varied among subjects (Nelson and Chen, 2008).

Overduin and Servos (2004) studied finger representation in BAs 3b and 1. They found larger representations in BA 1. They hypothesized that the differences in the size of the representations between the 2 areas were due to different functional properties of each of the areas. BA 3b appeared to receive inputs from two receptor types (Meissner’s corpuscles and Merkel’s disks) and thus may have multiple functional maps of the fingers (Overduin and Servos, 2004).

Puckett et al. (2020) looked at finger somatotopy using a Bayesian pRF model. They noted that the little finger had larger pRFs than any other finger. The size of pRFs were also shown to increase along the anterior-to-posterior axis. This supports findings that showed decreasing finger specificity moving posterior in S1. They also found that the index finger had significantly larger cortical representation than the other fingers studied (middle, index, and little finger) (Puckett et al., 2020).

Sanchez-Panchuelo et al. (2010) found activation to be located in the posterior aspect of the CS and the crown of the PG. Stimulation of the finger tips activated mainly the rostral bank of the postcentral sulcus (BA3b). Fingers were represented in an orderly way with the thumb being most inferior and lateral and the other 4 digits being represented in increasingly superior and medial locations (Sanchez-Panchuelo et al., 2010).

Sánchez-Panchuelo et al. (2014) found within-finger map reversals at the boundaries between all four areas of S1. This result indicates that instead of each phalanx of a finger being located in the same place in each area, the order reverses after crossing each border. That is, within finger representations, individual phalanges are not ordered in the same manner in each area (base to tip) but rather change the order after crossing the border between the areas. Specifically, BA3a is ordered “base to tip,” BA3b “tip to base,” BA1 “base to tip,” and BA2 “tip to base.” They also found a representation of all stimulated fingers (index, middle, and ring) in BA3a which is unique compared to other studies (Sánchez-Panchuelo et al., 2014).

Results from Sanchez Panchuelo et al. (2018) showed that the activation pattern of the digits falls almost entirely along the posterior CS and the anterior superior PG. The independent paradigm used in this study showed an orderly somatotopic organization in the primary somatosensory cortex but no such organization in the secondary somatosensory cortex (S2). Furthermore, overlap between adjacent digits was significantly larger in S2, however, all regions of interest located in S1 also showed an overlap. Findings derived from the traveling wave paradigm showed a lateral-to-medial and inferior-to-superior organization of the digits from the thumb to the little finger (Sanchez Panchuelo et al., 2018).

Schellekens et al. (2018) used Gaussian pRF model to study the finger representation in the primary motor (M1) and somatosensory cortex (S1). They found that pRF were significantly smaller in S1 compared to M1 which suggests that S1 processes information in greater detail even during motor movements. They also found that many neural populations in both cortices respond to several different finger movements suggesting an overlap between digits in both areas. However, the study did not investigate the different areas of S1 (BA3a, BA3b, BA1, and BA2) and therefore, we could not draw any conclusions on how the finger overlap differs in S1 Schellekens et al. (2018).

Schellekens et al. (2021) looked at pRF and how they varied across different areas of S1. They found that pRF for individual fingers increased in size posteriorly from BA3b. There was significantly less specific somatotopy in BA2 compared to BA3b and BA1. Furthermore, they examined the hierarchical order in which S1 was organized based on the time-to-peak of the hemodynamic response function (HRF). Results indicated that the response in BA3b was 0.5 s faster than in other areas (Schellekens et al., 2021).

Schweisfurth et al. (2014) investigated all phalanges and finger bases of the fingers on the right hand (dominant hand). They found that all the phalanges followed the well-established medial-to-lateral organization moving from D1 to D5. However, these results were not replicated for the finger bases (Schweisfurth et al., 2014).

Schweisfurth et al. (2018) looked at the differences between the somatotopy of dominant and non-dominant hands and found that D1 had the largest cortical representation of all the studied fingers on both hands. They also found that there were no significant differences in location of the fingers nor in the order in which the fingers were organized in the somatosensory cortex. However, they observed that two non-significant trends–left hand (non-dominant) D4 and D5 were more posteriorly located compared to the right hand D4 and D5. Additionally, the Euclidean distance of these two digits to the left hand D1 was larger than those on the right hand. These results introduce a potential factor that should be controlled for in future research (Schweisfurth et al., 2018).

Results of Schweizer et al. (2008) showed that digit representations in the human somatosensory cortex were found to be almost exclusively on the posterior wall of the CS which corresponds to BA 3b.

To study the differentiation of human somatosensory cortices, Stringer et al. used air puffs to stimulate individual digits of the participants. Findings revealed discrete single-digit responses in an area along the posterior bank of the CS corresponding to area 3b as well as in an area along the crest of the PG corresponding to area 1. This study also found a significantly greater magnification factor for all digits in BA3b compared to BA1 (Stringer et al., 2011).

Wang et al. (2021) found an orderly (D1–D5) representation of the fingers in the PG. The overlap between adjacent fingers decreased with increasing digit distance. They also indicated larger pRF sizes in BA1 and BA2 compared to BA3a and BA3b. Compared to findings in the right hand (Martuzzi et al., 2014), they did not find a significantly larger representation of the thumb in S1 Wang et al. (2021).

## Discussion

In this review, we examined the localization of the finger representations reported in different human fMRI studies. We focused on the spatial overlap between fingers, distance between D1 and D5, and cortical magnification findings. However, our review did not examine the activation strength of individual digits across the different areas. The measurement of activation strength can be deduced from the amount of overlap and distance between the digits in the different areas of the primary somatosensory cortex. Areas that show more overlap and smaller distance between adjacent digits have lower activation strength than those that are more finger specific (less overlap and bigger distance).

The most important variation in the fMRI parameters was the magnetic field strength. Higher field strength improves signal to noise ratio and spatial resolution. And while higher field strengths exacerbate fMRI artifacts, the primary somatosensory cortex fortuitously lies in an easy to image region for MRI. As spatial resolution is particularly important for these studies, the authors suggest that future studies should use field strengths of 7T or greater when available to achieve as accurate results as possible.

The reviewed studies also varied in the duration of stimulation. This could cause differences in the results due to habituation. However, none of the studies exceeded 35 s of continuous stimulation which, in our opinion, controls for this phenomenon. Most of the studies also randomized the order in which the digits were stimulated to prevent additional effects of expectation and prediction.

### Localization

Despite the different methods used in the reviewed literature, finger maps were predominantly located in BA3b, BA2, and BA1 showing a lateral-to-medial and inferior-to-posterior organization from digit 1 to digit 5 in the cortex, in all studies besides Gelnar et al. (1998) who did not find simple lateral-to-medial organization. This finding has been consistent across many studies conducted over more than 2 decades (Maldjian et al., 1999; Kurth et al., 2000; Hlustík et al., 2001; Overduin and Servos, 2004; Nelson and Chen, 2008; Schweizer et al., 2008; Sanchez-Panchuelo et al., 2010; Stringer et al., 2011; Besle et al., 2013; Martuzzi et al., 2014; Sánchez-Panchuelo et al., 2014; Schweisfurth et al., 2014, 2018; Kolasinski et al., 2016; Sanchez Panchuelo et al., 2018; Schellekens et al., 2018, 2021; Puckett et al., 2020; Arbuckle et al., 2021; Wang et al., 2021) and, thus, the authors feel that fMRI can be considered a reliable tool for somatosensory mapping research of the fingers at the group level. There remain some variations in the results that may be caused by differences in analysis methods or data acquisition parameters. For example, even though Sanchez-Panchuelo et al. (2012) showed that the distal phalanx responds most reliably to stimulation, multiple other studies stimulated all phalanges and did not report this effect.

Unique findings were also shown in Sanchez-Panchuelo et al. (2012), Sánchez-Panchuelo et al. (2014), and Wang et al. (2021) which revealed an additional activation in BA3a. This finding may have resulted from experiment specific regions of interest definitions or applied analysis and modeling. As suggested by studies in non-human primates, BA3a is involved in motor production and processing of kinesthetic afferents rather than cutaneous inputs (Kaas, 1993; Huffman and Krubitzer, 2001; Mountcastle, 2003).

Another unique finding was observed in Nelson and Chen (2008) which suggested that BA1 can be divided into 2 sub-areas–BA1 superior (BA1s) and BA1 inferior (BA1i). These two areas showed distinct activation with BA1s showing anterior-posterior, medial-lateral, and inferior-superior somatotopy while BA1i did not show any finger specificity and was activated after each digit stimulation. To our knowledge, these are the only findings in the current literature suggesting such division. Activation is BA1i could have been caused by a nearby vessel pointed out in previous work (Schweisfurth et al., 2018).

### Digit Distance

The somatotopic map of the hand occupies the largest space in BA3b as supported by the findings of D1–D5 distances across the primary somatosensory cortex (Nelson and Chen, 2008; Stringer et al., 2011; Martuzzi et al., 2014; Schweisfurth et al., 2014, 2018). Moving from D1 to D5, the distance between adjacent fingers decreases as reported by Kolasinski et al. (2016), and partly supported also by Schweisfurth et al. (2018). The medial-to-lateral model of finger representation has been supported by multiple studies showing increasing Euclidean distance between D1 and other digits (Schweisfurth et al., 2018, 2014; Wang et al., 2021). Additionally, Puckett et al. (2020) found that the distance between adjacent fingers decreases with the increasing pRF size of the fingers.

### Cortical Magnification

Some of the reviewed studies also discussed cortical magnification of the fingers in the primary somatosensory cortex, largely in BA3b. Cortical magnification can be described as the relative size of cortex activated based upon the relative receptive field size of the stimulated area (Cohen, 2011). Receptive field size and cortical magnification are inversely proportional and, therefore, smaller receptive field sizes yield larger cortical magnification factors (Harvey and Dumoulin, 2011). Studies that considered cortical magnification factors for individual digits revealed the smallest receptive field and largest cortical magnification for the thumb and the largest receptive field and smallest cortical magnification for the ring and little fingers (Maldjian et al., 1999; Overduin and Servos, 2004; Schweizer et al., 2008; Sanchez-Panchuelo et al., 2010; Martuzzi et al., 2014; Schweisfurth et al., 2018; Schellekens et al., 2021). In support of the previous results suggesting that the most used finger has the largest cortical magnification, Puckett et al. (2020) found that D2 had the largest cortical representation compared to other studied digits (D3, D4, and D5). Stringer et al. (2011) did not discuss cortical magnification of individual digits, but found that there was a significantly greater magnification factor in BA3b compared to BA1.

### Overlap

Overlap between adjacent fingers was examined across different studies. Even though only one finger was stimulated at a time, the fingers seem to share a certain, yet varying, amount of cortical space across S1. The exact amount of overlap was not consistent, but all studies agreed that the overlap generally increases moving posterior from BA3b. This shows that BA3b is likely the initial receiver of somatosensory information arriving at the somatosensory cortex. This is further supported by Schellekens et al. (2021) who found that BA3b hemodynamic response function is 0.5 s faster than in the other two areas (Schellekens et al., 2018). The most plausible explanation for more spatial overlap between adjacent digits in BAs 1 and 2 compared to BA3b is the size of the cell receptive fields. Based on the research in non-human primates, the size of receptive fields extends over multiple fingers in BAs 1 and 2 (Iwamura et al., 1983, 1985). This hypothesis is strengthened by findings of Schellekens et al. (2021) who reported that pRFs are increasing going posterior from BA3b. pRFs can be understood as a cumulative response of neural cells contained within a voxel (Dumoulin and Wandell, 2008). Interestingly, some studies reported BA3b being more finger specific compared to other studies (Schweizer et al., 2008; Martuzzi et al., 2014; Arbuckle et al., 2021). The differences in reported overlap may also be due to the variabilities between the experimental designs/analysis, stimulation duration, smoothing, or MRI field strength in the studies as discussed in Tables 1, 2. Another hypothesis is that the type of stimulation results in variation of the finger overlap observed in the studies. This particular hypothesis is not directly addressed by the literature, though, since there are no studies that specifically compare different types of stimulation. It is, however, well established that BAs 3b, 1, and 2 are specialized for different types of receptors–Meissner’s corpuscles, Pacinian corpuscles, Merkel’s disks, and Ruffini’s corpuscles. Meissner’s corpuscles respond to dynamic skin deformation and low-frequency vibration, Pacinian corpuscles are sensitive to high-frequency stimuli, Ruffini corpuscles are responsive to low-frequency vibration or pressure, and Merkel’s discs are sensitive to light touch and transmit spatial structure of objects (Mountcastle, 1998; Johnson et al., 2000). In cynomolgus monkeys BA 3 is activated by pressure, vibration, and tactile stimulus, BA 2 is activated by pressure stimulus, and BA 1 is activated mostly by vibrotactile stimulus (Sur, 1980). This is consistent with Overduin and Servos (2004) who show in humans that BA3b may include multiple functional maps of the digits since it is activated by 2 different stimuli that are preferential for 2 different types of receptors. Activation of different sub-populations of neurons in the somatosensory cortex by stimuli with different sensory properties could result in differences in localization, the amount of activation, and variations in activation overlap.

Overlap as a function of digit varied across studies. Maps of digits 4 and 5 demonstrated the most overlap in Martuzzi et al. (2014) and Arbuckle et al. (2021). However, Besle et al. (2013), reported that the most overlap exists between digits 3 and 4. This might be explained by the natural “pairing” of the fingers by function as it has been shown to have an effect on mapping (Ejaz et al., 2015) though it is unclear why the strongest pairing would vary across studies.

Another study that suggested the effect of usage on the results at the intra digit level was Schweisfurth et al. (2014). They demonstrated that only phalanges of D5 showed a within-finger map stability across subjects suggesting an effect of daily usage on the location and size of individual phalanx representations (Schweisfurth et al., 2014).

The most important variation in the fMRI parameters is the magnetic field strength. Higher field strength improves signal to noise ratio and spatial resolution. And while higher field strengths exacerbate fMRI artifacts, the primary somatosensory cortex fortuitously lies in an easy to image region for MRI. As spatial resolution is particularly important for these studies, the authors suggest that future studies should use field strengths of 7T or greater when available to achieve as accurate results as possible.

The reviewed studies also varied in the duration of stimulation. This could cause differences in the results due to habituation. However, none of the studies exceeded 35 s of continuous stimulation which, in our opinion, controls for this phenomenon. Most of the studies also randomized the order in which the digits were stimulated to prevent additional effects of expectation and prediction.

## Conclusion

Despite the variation in MRI parameters, experimental designs, and stimuli used in the reviewed literature, finger maps were predominantly located in BA3b, BA2, and BA1 showing a lateral-to-medial and inferior-to-posterior organization from digit 1 to digit 5 in the cortex. This finding has been consistent across many studies conducted over more than 2 decades and is consistent with the non-human primate literature. Thus, the authors feel that fMRI can be considered a reliable tool for somatosensory mapping research of the fingers at the group level. There remain, however, some variations in the results that may be caused by differences in analysis methods or data acquisition parameters. The variation in reported results due to MRI parameters are of less inherent interest than the variations due to experimental design and stimulus properties as the latter effects may reveal information about the representation and processing of sensory information in the human brain.

Based on the reviewed studies, we suggest that BA3b could be considered to be functionally distinct from BA 1, 2, and 3a purely on the basis of fMRI studies as BA3b shows the greater cortical magnification, larger D1-D5 distance, and a faster BOLD response compared to BA 1, 2, 3a. In addition these results suggest that BA3b should be considered the primary somatosensory cortex with BA 1, 2, and 3a being the supplementary cortex.

The authors recognize an important limitation of this review–some studies did not specifically focus on the overlap between fingers and therefore, it may be under-reported. We feel that a comparison of the effects of different types of stimulation on the amount of overlap between adjacent digits in different areas is needed to better explain the inconsistencies discussed previously. The third area that we suggest future research should focus on is examining which finger pairs have the most overlap between each other and how that varies based on self-reported frequency of use. To our knowledge, only one study directly measured the intrasubject reproducibility of the maps over an extended period of time (Kolasinski et al., 2016) and partially also Arbuckle et al. (2021). Thus, there should be more investigation into the intrasubject variability of the size, localization, and strength of activation of different maps over time.

## Author Contributions

MB conceived the initial ideas and edited the manuscript. MB, DJ, KT, DP, and MH performed the literature search. KT, DJ, and DP wrote the manuscript and contributed equally to the manuscript. All authors contributed to the article and approved the submitted version.

## Conflict of Interest

The authors declare that the research was conducted in the absence of any commercial or financial relationships that could be construed as a potential conflict of interest.

## Publisher’s Note

All claims expressed in this article are solely those of the authors and do not necessarily represent those of their affiliated organizations, or those of the publisher, the editors and the reviewers. Any product that may be evaluated in this article, or claim that may be made by its manufacturer, is not guaranteed or endorsed by the publisher.
